# Investigation of Key Interventions for Shigellosis Outbreak Control in China

**DOI:** 10.1371/journal.pone.0095006

**Published:** 2014-04-15

**Authors:** Tianmu Chen, Ross Ka-kit Leung, Zi Zhou, Ruchun Liu, Xixing Zhang, Lijie Zhang

**Affiliations:** 1 Office for Disease Control and Emergency Response, Changsha Center for Disease Control and Prevention, Changsha, The People’s Republic of China; 2 Stanley Ho Centre for Emerging Infectious Diseases, The Chinese University of Hong Kong, Shatin, New Territories, Hong Kong SAR, The People’s Republic of China; 3 School of Public Health, Xiamen University, Xiamen, The People’s Republic of China; 4 Chinese Field Epidemiology Training Program, Chinese Center for Disease Control and Prevention, Beijing, The People’s Republic of China; The University of Tokyo, Japan

## Abstract

Shigellosis is a major public health concern in China, where waterborne disease outbreaks are common. Shigellosis-containing strategies, mostly single or multiple interventions, are implemented by primary-level health departments. Systematic assessment of the effectiveness of these measures is scarce. To estimate the efficacy of commonly used intervention strategies, we developed a Susceptible–Exposed–Infectious/Asymptomatic–Recovered–Water model. No intervention was predicted to result in a total attack rate (TAR) of 90% of the affected population (95% confidence interval [CI]: 86.65–92.80) and duration of outbreak (DO) of 89 days, and the use of single-intervention strategies can be futile or even counter-productive. Prophylactics and water disinfection did not improve TAR or DO. School closure for up to 3 weeks did not help but only increased DO. Isolation alone significantly increased DO. Only antibiotics treatment could shorten the DO to 35 days with TAR unaffected. We observed that these intervention effects were additive when in combined usage under most circumstances. Combined intervention “Isolation+antibiotics+prophylactics+water disinfection” was predicted to result in the lowest TAR (41.9%, 95%CI: 36.97–47.04%) and shortest DO (28 days). Our actual Shigellosis control implementation that also included school closure for 1 week, attained comparable results and the modeling produced an epidemic curve of Shigellosis highly similar to our actual outbreak data. This lends a strong support to the reality of our model that provides a possible reference for public health professionals to evaluate their strategies towards Shigellosis control.

## Introduction

Shigellosis (bacillary dysentery), the result of infection with *Shigella*, is an enteric infectious disease responsible for approximately 1,100,000 deaths per year worldwide [1]. As approximately two-thirds of those who die from shigellosis are children under 5 years of age, it is one of the most common diarrhea-related causes of morbidity and mortality in children in developing countries [2]. Shigellosis epidemics usually occur in areas with crowding and poor sanitary conditions, where person-to-person transmission or contamination of food or water by the organism is common [3]–[8]. In China, many private wells supplying water to schools are built in close proximity to sources of pollution, including toilets, septic tanks, sewer ditches, and lakes and ponds into which sewage is discharged. As water from these wells is often not treated before being piped into schools, waterborne outbreaks of *Shigella* frequently occur [6], with devastating effects on students, their families, and schools.

Many outbreak control strategies developed by primary-level health departments in China are empirically-driven. This can be attributed to a lack of data regarding the rate of morbidity in the absence of intervention, making it difficult to estimate whether the efficacy of a single or combined intervention could be decreased if implemented using traditional methods. In these circumstances, researchers often perform mathematical modeling to estimate the total attack rate (TAR), an indicator of the extent of an outbreak [9]–[13]. A bacillary dysentery model with seasonal fluctuation was formulated and studied by Bai et al. [14], in which a simple Susceptible–Infectious–Recovered–Susceptible framework was employed that could not clarify the person–water–person transmission pathways.

Fortunately, a waterborne pathogen model termed the Susceptible–Infectious–Recovered–Water (SIRW) model can be used to examine disease outbreaks that occur via multiple transmission pathways [15], such as shigellosis. The SIRW model is a simple ordinary differential equation model that extends the classic SIR framework by adding a compartment (W) that tracks the pathogen concentration in water. Infected individuals shed the pathogen into water compartments, and new infections arise both through exposure to contaminated water as well as by the classic SIR person–person transmission pathway. Combining the characteristics of the SIRW model with those of shigellosis, we developed a Susceptible–Exposed–Infectious/Asymptomatic–Recovered–Water (SEIARW) model to examine the efficacy of different intervention strategies in controlling an outbreak of shigellosis at a primary school in Changsha City, China.

## Materials and Methods

### Ethics Statement

Each shigellosis case was required to be notified for epidemiologic surveillance. These surveillance data were used in this study, without the need for the collection of additional information (e.g. demographics) for the research. This study was approved by Medical Ethics Committee of Changsha Center for Disease Control and Prevention. Consent requirement, either verbal or written, was waived by Medical Ethics Committee of Changsha Center for Disease Control and Prevention Center for Disease Control and Prevention on the following grounds: (1) only anonymized records were used without the need for direct involvement nor active participation of patients; (2) neither medical intervention nor biological samples were involved; (3) study procedures and results would not affect clinical management of patients in any form.

### Data Collection and Analysis

On October 19, 2012, a local branch of the Center for Disease Control and Prevention (CDC) reported an outbreak of shigellosis in a primary school in a rural area. As the longest incubation period is 9 days, subsequent immediate investigation between October 3 and 31, reflecting identification of the first and final cases on October 11 and 23, respectively, permitted data collection for 134 cases. However, no case was found to be chronic or fatal, and 7 anal swab samples were collected from healthy individuals from the school, and no culture was positive for *Shigella sonnei*. The 2 primary transmission routes were identified as person–to-person and person–water–person; *Shigella sonnei* was identified as the pathogen responsible for the outbreak. The definition of a case in this investigation followed that specified in “Diagnostic criteria for bacillary and amoebic dysentery (WS 287–2008),” a report published by the Ministry of Health of China in 2008 based on a shigellosis outbreak investigation conducted by He et al. [6]. A probable case of shigellosis was identified in a teacher or a student of the primary school or in a resident who lived in close proximity to the school between October 5 and 28, if the affected individual experienced diarrhea ≥3 times per day in addition to ≥1 of the following symptoms: fever ≥37.5°C, vomiting, and/or abdominal pain. A confirmed case was identified when culture confirmation of *Shigella* infection was obtained from a stool specimen or rectal swab from an individual who had been identified as a suspected case.

This outbreak was used as an example for modeling the use of combined strategies for containing a shigellosis outbreak, including 4 major measures (case isolation, medical intervention, school closure, and water disinfection) and 3 supplementary measures (environmental disinfection, health education, and hand hygiene). As the supplementary measures were difficult to quantify, a combination of the resulting in 22 strategy options, including single and combined-intervention strategies was simulated.

We collected the basic information of the school and the data of cases. The former included the number of students and faculty and staff, and the number of classes. The later included name, sex, age,class of each case, and the date of illness onset, the date of medical intervention implemented, the date of recovered, and the symptoms (such as fever, headache or dizziness, times of diarrhea per day, stool texture, abdominal pain, vomit, etc.) of each case. From this data, we can analyze the temporal distribution of each case and the infectious period with medical intervention or not.

### No Intervention

A deterministic model was developed on the basis of the following conditions:

1. Transmission occurs via either a person–person or a person–water–person route.
2. Although fatal cases (albeit a low number) have been identified in previous shigellosis epidemic, it was not identified in the outbreak investigated here. Thus, fatal cases were not included in the model.
3. We also considered asymptomatic cases in the model since asymptomatic carriers have been identified in previous outbreaks.
4. Infection during an outbreak confers permanent immunity.
5. The transmission of shigellosis during a school outbreak occurs within a closed system, defined as a system with no migration in or out; adjustment for births and natural deaths was not included in the model.
6. The county in which the affected school is located contains 33 towns and a population of more than 1.37 million. Despite these numbers, only 8 cases of shigellosis were reported among individuals who were not affiliated with the school, indicating a low probability of infection among them. Thus, the model did not consider the probability of infection in the wider community.

**Figure 1:**
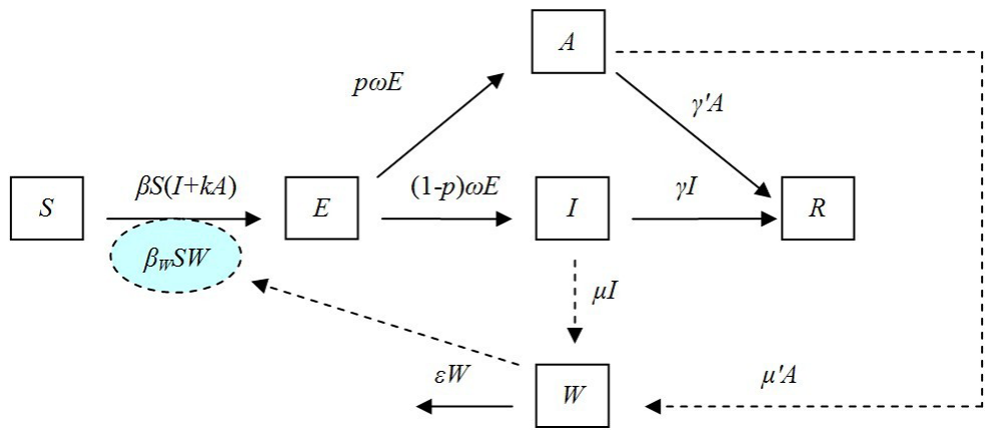
Flowchart of development of the SEIARW model (1).

Figure 1 depicts the flow diagram for the development of the SEIARW model, where individuals are characterized according to their epidemiological status as susceptible (*S*), exposed (*E*, infected but not yet fully contagious), infectious (*I*), asymptomatic (*A*), and recovered (*R*); *W* denotes the reservoir (water) compartment. The definitions of the 6 epidemiological classes are summarized in Table 1.

**Table 1:** Variables within the SEIARW model (1).

Variable	Description	Unit
*S*	Susceptible individual density	Individuals·km^−2^
*E*	Exposed individual density	Individuals·km^−2^
*I*	Infectious individual density	Individuals·km^−2^
*A*	Asymptomatic individual density	Individuals·km^−2^
*R*	Recovered/removed individual density	Individuals·km^−2^
*N*	Total population density	Individuals·km^−2^
*W*	Pathogen concentration in water reservoir	Cells·mL^−3^

As illustrated in Figure 1, susceptible individuals become infected (i.e., move from *S* to *E*) by contact with either infected/asymptomatic individuals or contaminated water at rates of *βSI*, *βkSA* and *β_W_SW* respectively, where *β* and *β_W_* are the probability of transmission per contact, *k* is the relative transmissibility of asymptomatic to symptomatic individuals. As exposed individuals become infectious after an incubation period, they move from *E* to *I* at a rate of (1−*p*)*ωE* and *E* to *A* at a rate of *pωE*, where 1/*ω* is the incubation period of the disease and *p* is the proportion of asymptomatic individuals. After the infectious period has passed, infectious and asymptomatic individuals may move to *R* at a rate of *γI* and *γ’A* respectively, where 1/*γ* and 1/*γ’* are the infectious period of the *I* and *A*. Infectious and asymptomatic individuals can in turn contaminate the water compartment by shedding the pathogen into *W* at a shedding rate of *μI* and *μ’A*, where *μ* and *μ’* are the shedding coefficients. The pathogen in *W* will subsequently leave the water compartment at a rate of *εW*, where 1/*ε* is the lifetime of the pathogen. The corresponding model equations are as follows:
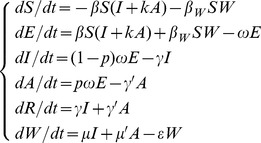
(1)


It would be instructive to consider a rescaling of model (1) using dimensionless variables. If *N* is assumed to denote the total population size and *s* = *S/N*, *e* = *E/N*, *i* = *I/N*, *a* = *I/A*, *r* = *R/N*, *w* = *εW/μN*, *μ’ = cμ*, *b* = *βN*, and *b_W_* = *μβ_W_N/ε*, the following rescaled model can be developed:
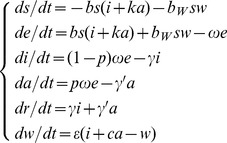
(2)


As the SEIAR model assumes that transmission occurs via daily contact, it assumes that transmission occurs solely via the person–person route. The corresponding model equation is thus:
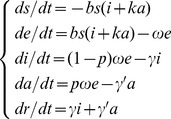
(3)


### Case Isolation

In practice, milder shigellosis cases were requested to stay home. Dedicated staff paid visits to ensure adherence, hygiene and proper disinfection. More severe cases were hospitalized and isolated. Both cases received a full course of antibiotics treatment and were discharged two days since the day they have been free of symptoms. In the case isolation model, neither the reservoir-to-person nor the person–to-person routes are viable means of transmission. Nevertheless, individuals in compartment *S* could become infected via the reservoir–to-person and asymptomatic-susceptible routes, leading to development of the following rescaled model:
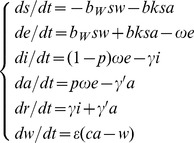
(4)


### Medical Intervention

**Figure 2:**
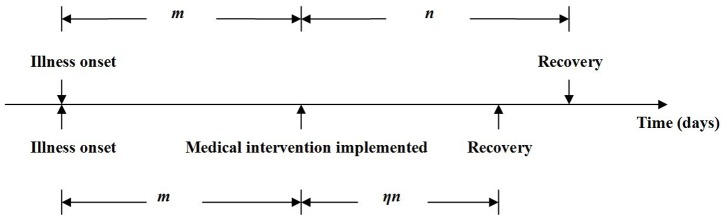
Infectious period of a typical shigellosis case with or without medical intervention. *m*+*n*: Infectious period with no medical intervention; *m*+*ηn*: infectious period with partial medical intervention, which is equivalent to part of the infectious period with no medical intervention (*m*) and part of the infectious period with medical intervention (*ηn*); *η*(*m*+*n*): infectious period with a medical intervention that had been implemented on the date of illness onset.

Medical interventions including therapeutics and prophylactic, were both simulated in our model. For therapeutics, several antibiotics, including ampicillin-sulbactam sodium, ceftazidime pentahydrate, cefixime, and cefaclor, had all been used in exactly the same manner for different individuals during the outbreak; we used drug sensitivity tests to determine their effectiveness against *S. sonnei*. If a case of shigellosis is treated with a standard course of antibiotics from the date of illness onset, the mean infectious period can be reduced by *η*, which ranges from 0 to 1 (Figure 2).

For prophylactic, berberine hydrochloride was targeted at individuals other than recovered (*R*) or infectious (*I*), so susceptible (*S*), exposed (*E*), asymptomatic (*A*) individuals were all targeted. We assumed that given a standard course (3 days) of berberine hydrochloride, a susceptible individual cannot be infected by *S. sonnei* for 3 days, an exposed individual will become recovered (*R*) directly, and the transmissibility of asymptomatic individual will be discounted by *f*, where *f* ranges from 0 to 1.

### Water Disinfection

We have been following the guidelines of “Technical Standard For Disinfection (The 2002 Edition)” issued by the Chinese National Health and Family Planning Commission and chlorine-releasing agents, such as chlorinated lime and calcium hypochlorite have been being used for disinfection. According to a previous study conducted by Wu et al [16], no *S*. *sonnei* was found in chlorinated water. Therefore, the variables *W*, *ε*, and *b_W_* are removed from the water disinfection model.

### School Closure

During a school closure, all the people in a school return home. Person–to-person and reservoir–to-person contacts are severed, making both *b* and *b_W_* become zero in effect in the school closure model. We simulated the school closure of 1, 2, or 3 weeks, to examine the effects.

### Combined-Intervention Strategies

We simulated the following 17 combined interventions to examine their impact: AW, antibiotics+water disinfection; IA, isolation+antibiotics; IW, isolation+water disinfection; S_1w_I, school closure (1 week)+isolation; S_1w_W, school closure (1 week)+water disinfection; S_1w_A, school closure (1 week)+antibiotics; APW, antibiotics+prophylactic+water disinfection; IAP, isolation+antibiotics+prophylactic; IPW, isolation+prophylactic+water disinfection; S_1w_IP, school closure (1 week)+isolation+prophylactic; S_1w_PW, school closure (1 week)+prophylactic+water disinfection; S_1w_AP, school closure (1 week)+antibiotics+prophylactic; IAPW, isolation+antibiotics+prophylactic+water disinfection; S_1w_IPW, school closure (1 week)+isolation+prophylactic+water disinfection; S_1w_IAP, school closure (1 week)+isolation+antibiotics+prophylactic; S_1w_APW, school closure (1 week)+antibiotics+prophylactic+water disinfection; S_1w_IAPW, school closure (1 week)+isolation+antibiotics+prophylactic+water disinfection.

### Estimation of Parameters

Of *b*, *b_W_*, *k*, *ω*, *p*, *γ, γ’*, *c*, *ε*, *f*, and *η*, the 11 parameters in the model (Table 2), *γ* and *η* could be estimated by reviewing data regarding the outbreak. According to the reports from the Department of Health, Victorian State Government, North Dakota Department of Health Division of Disease Control and New Mexico Department of Health Epidemiology and Response Division Infectious Disease Epidemiology Bureau, asymptomatic individuals can shed the bacteria for at least 4 weeks. Here we simulated 5 weeks in our model, thus *γ’* = 0.0286.

**Table 2:** Parameter definitions and values.

Parameter	Description	Unit	Value	Method
*β*	Person–to-person contact rate	km^2^·individuals^−1^·day^−1^	–	–
*b*	Scaled person– to-person contact rate	day^−1^	1.1240	Curve fitting
*β_W_*	Reservoir– to-person contact rate	mL^3^·cells^−1^·day^−1^	–	–
*b_W_*	Scaled reservoir– to-person contact rate	day^−1^	1.1289	Curve fitting
*k*	Relative transmissibility rate of asymptomaticto symptomatic individuals	1	0.3125	Analysis of outbreak data
*ω*	Incubation relative rate	day^−1^	1	Curve fitting
*p*	Proportion of the asymptomatic	1	0.1	References[20]–[22]
*γ*	Recovery rate of the infectious	day^−1^	0.0741	Analysis of outbreak data
*γ'*	Recovery rate of the asymptomatic	day^−1^	0.0286	See text
*ε*	Pathogen lifetime relative rate	day^−1^	0.6931	Calculation using Equation (5)
*μ*	Person–to-reservoir contact rate (“shedding”by Infectious)	cells·mL^−3^·day^−1^·km^2^·individuals^−1^	–	–
*μ'*	Person–to-reservoir contact rate (“shedding”by Asymptomatic)	cells·mL^−3^·day^−1^·km^2^·individuals^−1^	–	–
*c*	Shedding rate of the asymptomatic comparingto the infectious	1	0.3125	Analysis of outbreak data
*η*	Efficacy of medical intervention [antibiotics]	1	0.3111	Analysis of outbreak data
*f*	Efficacy of medical intervention [prophylactic]	1	5.7221×10^–7^	Curve fitting

Several studies have found that although *S. sonnei* can survive from several to 170 days and can even grow well in food [17], [18], its die-off rate (half-time) in well water is only 24.5 h [19], meaning that only 50% of the *S. sonnei* present at the beginning of a study period (*t* = 0) remains after 1 day (*t* = 1). According to the SEIARW model, the die-off rate of *S. sonnei* in well water can be represented by the following equation:
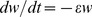
(5)


Thus, *ε* = 0.6931 under the conditions described above.

In our data, a typical case of diarrhea was about 3.2 times (range 3–12 times) per day but an asymptomatic individual only shedding stool once per day. Thus *μ’ = cμ*, c = 0.3125. Due to reduction of shedding frequency, the relative transmissibility of asymptomatic individual (*k*) was modeled to be a reduced quantity (0.3125). The proportions of asymptomatic individuals were reported to range from 0.0037 to 0.27 [20]–[22]. We set *p* = 0.1 in SEIARW model.

Although the incubation period of shigellosis ranges from 7 h to 9 days and is typically 1 to 3 days [23], [24], *ω* cannot be obtained from analysis of the collected data. The parameters *b* and *ω* were therefore estimated by curve fitting using outbreak data collected from October 11 to 15 in the SEIAR model, the parameter *b_W_* using outbreak data collected from October 16 to 19 in the SEIARW model, and the parameter *f* using outbreak data collected from October 19 to 28 in the SEIARW model.

### Strategy Assessment Indicators

We estimated TAR and duration of outbreak (DO) to assess the efficacy of the strategies for controlling the outbreak (Table 3). Epidemic curves were compared to evaluate the efficacy of using the containing strategies as compared to no intervention and to the actual combined strategies implemented by the health and education departments.

**Table 3:** Results of simulation of effectiveness of shigellosis interventions implemented on October 19.

	TAR (%)		
Intervention	%	95% CI	DO (days)
None	90.0	86.65–92.79	89
Antibiotics	90.0	86.65–92.79	35
Prophylactics	90.0	86.65–92.79	89
Water disinfection	90.0	86.65–92.79	90
Isolation	85.3	81.20–88.48	147
School closure			
1 week	90.0	86.65–92.79	94
2 weeks	90.0	86.65–92.79	100
3 weeks	90.0	86.65–92.79	107
AW	89.6	86.03–92.32	37
IA	84.5	80.30–87.74	93
IW	70.8	66.10–75.38	180
S1wI	83.4	79.41–87.01	175
S1wW	90.0	86.65–92.79	95
S1wA	90.0	86.65–92.79	37
APW	87.7	83.90–90.66	46
IAP	44.5	39.37–49.51	38
IPW	41.9	36.97–47.04	70
S1wIP	42.1	37.23–47.31	73
S1wPW	90	86.65–92.79	94
S1wAP	89.8	86.34–92.55	46
IAPW	41.9	36.97–47.04	28
S1wIPW	41.9	36.97–47.04	70
S1wIAP	41.9	36.97–47.04	28
S1wAPW	86.7	82.70–89.70	58
S1wIAPW	41.9	36.97–47.04	28

TAR, Total attack rate; CI, confidence interval as calculated by binomial distribution; DO, duration of outbreak.

### Simulation Methods

Berkeley Madonna 8.3.18 and Microsoft Office Excel 2003 software were employed for model simulation and figure development, respectively. The Runge-Kutta method of order 4 with the tolerance set at 0.001 was used to perform curve fitting. While the curve fit is in progress, Berkeley Madonna displays the root mean square (RMS) deviation between the data and best run so far.

### Sensitivity Analysis

Since two parameters, *p* and *γ'*, were estimated by references, there was some uncertainty about them which might impact the results of models we built. In our study, sensitivity was tested by varying the two parameters which were split into 1000 values ranging from 0.0037 to 0.27 and from 0.0036 to 0.0357 (which means asymptomatic individuals can shed the bacteria from 4 weeks to 40 weeks) respectively.

## Results

### Timing of Outbreak

**Figure 3:**
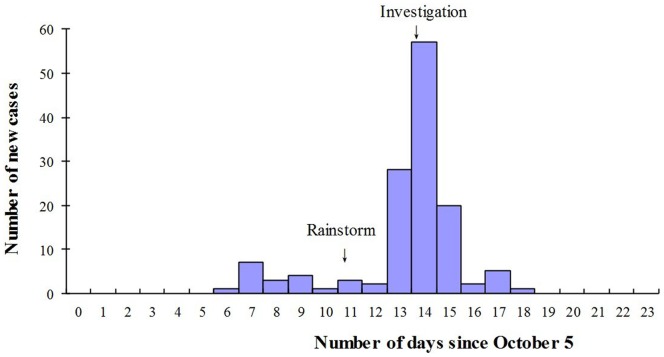
Temporal distribution of shigellosis new cases in a primary school since October 5.

Among the 348 students and 17 faculty and staff present at the school when the outbreak occurred, we identified 134 cases of shigellosis, of which 59 were suspected, 55 probable, and 20 confirmed, yielding a TAR of 36.71%. After the first case had been identified on October 11, the number of cases increased gradually, peaking between October 12 and 14, and then beginning to decrease on October 15. However, after a rainstorm on October 16 resulted in contamination of the shallow school well by sewage containing the feces of the cases, as identified using laboratory detection methods, the number of cases began to increase rapidly, peaking on October 19 before beginning to decrease after the local CDC initiated an investigation on the same day until the last case was identified on October 23. Field epidemiological study revealed that the 2 primary routes of transmission corresponded to the 2 epidemic peaks in terms of temporal distribution, with the first peak resulting from primarily person–to-person transmission, and the second from both person–water–person and person–to-person transmission (Figure 3).

**Figure 4:**
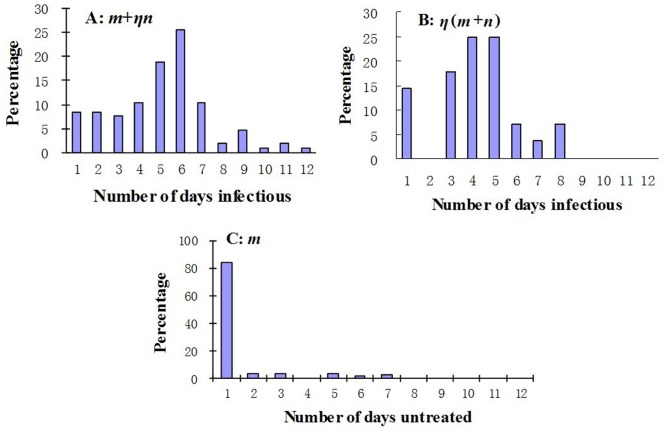
Infectious periods of all cases. A, Distribution of cases provided with fractional medical intervention (*m*+*ηn*) during infectious period; B, distribution of cases provided with combined intervention (*η*[*m*+*n*]) during infectious period; C, distribution of part of the infectious period with no medical intervention (*m*).

Analysis of all data collected revealed that the mean infectious period of all cases provided with partial medical intervention (*m*+*ηn*) was 5.1 (2.4 SD) days (range 1–12 days) (Figure 4A), with *m* = 1.3 (Figure 4C), whereas the mean infectious period of all cases with full intervention (*η*[*m*+*n*]) was 4.2 (1.9 SD) days (range 1–8 days) (Figure 4B). As no chronic cases were identified, the mean infectious period of all cases provided with no medical intervention (*m*+*n*) was 13.5 days, with *η* = 0.3111.

**Figure 5:**
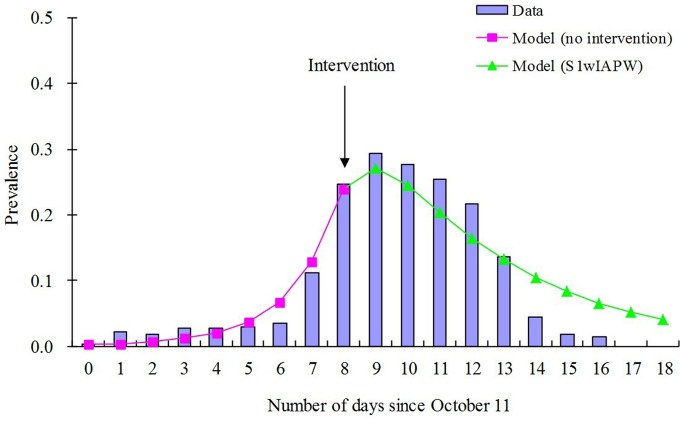
Curve fitting of data from the baseline of the outbreak simulation from October 11 to 29. Since the local CDC investigated and implemented the actual combined strategies at day 8 in this outbreak, SEIARW model with no intervention was employed for curve fitting during 0∼8 days (pink line), and SEIARW model with combined strategies of S1wIAPW was employed for curve fitting for the days thereafter (green line). Prevalence = *I*/*N* = *i*, where *I* is the infectious and *N* is the total number of persons.

Curve fitting analysis revealed that the daily prevalence of the outbreak fit the data to the greatest extent when *b* = 1.1240, *b_W_* = 1.1289, *ω* = 1, and *f* = 5.7221×10^–7^ (Figure 5). The model thus reproduced the typical epidemic curve observed for a shigellosis outbreak in a school population.

### Efficacy of Interventions

Table 3 lists the results regarding analysis of the efficacy of the 22 intervention strategies. In the absence of any intervention, the model predicted that all exposed individuals would become infected and sick, yielding a TAR of 90% (95% confidence interval [CI]: 86.65–92.79) and a DO of 89 days. We note that the TAR only includes observable cases (i.e. it does not include asymptomatic cases). Single-intervention options were predicted to result in high TAR. Specifically, strategy antibiotics had a TAR of 90% (95% CI: 86.65–92.79), but a relatively brief DO of 35 days. While strategy water disinfection also had a TAR of 90% (95% CI: 86.65–92.79), it had a much longer DO of 90 days. Likewise, the 3 school closure options (1 week, 2 weeks, and 3 weeks) all had a TAR of 90% (95% CI: 86.65–92.79) and a DO of 94, 100, and 107 days, respectively, all of which were longer than the DO of no intervention. Strategy prophylactics had no effects as if there was no intervention at all. Although strategy isolation was predicted to reduce the TAR slightly to 85.3% (95% CI: 81.20–88.48), the cost was a DO of 147 days.

Combined-intervention strategies that consist of only 2 single intervention options had little improvement over the single-intervention options. Among these 6 options, IW was the most effective, reducing the TAR to 70.8% (95% CI: 66.10–75.38) but a DO of 180 days, followed by, in descending order of efficacy, S_1w_I, IA, AW, S_1w_A, and S_1w_W. Combined-intervention strategies that consist of 3 single intervention options had better efficacy. Among these 6 options, IPW was the most effective, reducing the TAR to 41.9% (95% CI: 36.97–47.04) and the DO to 70 days, followed by S_1w_IP, IAP, APW, S_1w_AP, and S_1w_PW.

The 5 combined-intervention options that each included 4 or 5 different single options had the best efficacy. IAPW, S_1w_IAP, and S_1w_IAPW had almost the same efficacy, specifically a TAR of 41.9% (95% CI: 36.97–47.04) and a DO of only 28 days. Although S_1w_IPW had a similar TAR to IAPW, its DO was longer, and S_1w_APW had a longer DO and higher TAR than IAPW.

**Figure 6:**
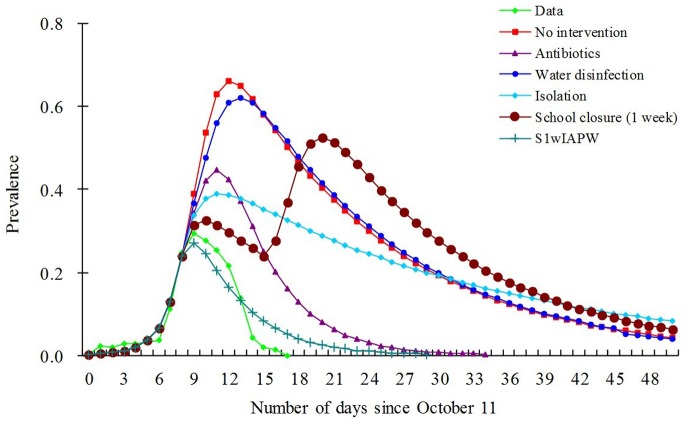
Results of simulation of efficacy of main intervention strategy options for controlling shigellosis outbreak. Prevalence = *I*/*N* = *i*, where *I* is the infectious and *N* is the total number of persons.

Figure 6 depicts the epidemic curve of the outbreak without intervention or with each intervention strategy. As can be observed, the curve of the single-intervention strategy water disinfection is most similar to that of the curve of no intervention; the curve of S_1w_IAPW is almost the same and most similar to the curve of the data of this outbreak in which actual combined strategies had been implemented.

### Sensitivity Analysis

**Figure 7:**
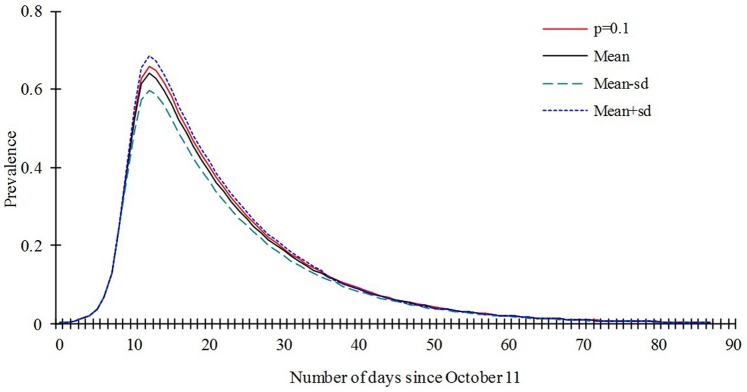
Sensitivity analysis to *p* based on the 1000 runs of the SEIARW model where *p* ranges from 0.0037 to 0.27.

**Figure 8:**
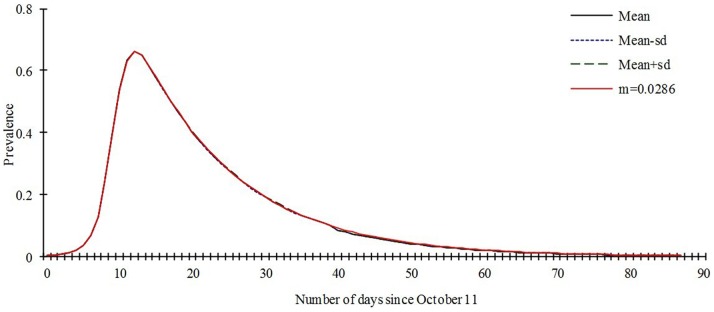
Sensitivity analysis to *γ'* based on the 1000 runs of the SEIARW model where *γ'* ranges from 0.0036 to 0.0357.

Our model is only slightly sensitive to the parameter *p*, the value which we set in our model (*p* = 0.1) lead to the same prevalence to the mean value of sensitivity analysis based on the 1000 of the model ran (Figure 7). The model is not sensitive to the parameter *γ'*, the prevalence are the same to the mean value, mean-sd, mean+sd, and the value we set (figure 8).

## Discussion

In China, cooperation between local health departments (i.e., local CDC branches), which develop outbreak control and intervention strategies, and education departments (i.e., schools), which implement the strategies, is necessary for controlling outbreaks, especially at the primary school level. It is crucial to determine whether these strategies are likely to be effective before adopted. To aid in fulfilling the need for such an estimation, we developed models to estimate the effectiveness of a variety of strategies in controlling an outbreak of shigellosis, yielding findings that will be of great importance for controlling future outbreaks.

Our results revealed that the effectiveness of single-intervention strategies in containing outbreaks would be limited, whereas that of combined-intervention strategies would be significantly greater. Specifically, the strategies IAPW, S_1w_IAP, and S_1w_IAPW, all of which had a TAR of 41.9% (95% CI: 36.97–47.04) and a DO of 28 days, thus reflecting the efficacy of the actual combined strategies most closely, would be the most effective.

Currently, school closure is the most common strategy used by primary-level health departments in China to control infectious disease outbreaks. However, this strategy has several negative effects on students, especially those who are about to graduate from primary or junior high school, their families, and the entire school. It has been demonstrated that closing schools during an epidemic may impose substantial costs on society, particularly the loss of productivity and the necessity of childcare, that far outweigh the cost savings in preventing infection [25]. Moreover, our results indicate that school closure, whether as a single strategy or in combination with other strategies, may not be effective, or may even exacerbate an outbreak. As our results indicate that the most effective strategy for controlling an outbreak of shigellosis is a combined strategy of case isolation, antibiotic administration, and water disinfection, this, rather than a school closure–based strategy, should be used to control future outbreaks. A similar observation was also identified in water disinfection, which was predicted to add marginal benefit in the shigellosis control. A single isolation intervention might do more harm than good. All these pieces of information point to the need for attention to careful design and evaluation of strategies before implementation.
